# Feasibility Study of the “HemoTypeSC” Test for the Rapid Screening of Sickle Cell Disease in Côte D'Ivoire

**DOI:** 10.1155/2021/8862039

**Published:** 2021-03-19

**Authors:** Jeannette Bassimbié Kakou Danho, Yao Nicaise Atiméré, Daouda Koné, Donafologo Daouda Yéo, Line Couitchéré

**Affiliations:** UFR Sciences Médicales d'Abidjan, Université Felix Houphouët Boigny, Abidjan, Côte d'Ivoire

## Abstract

Sickle cell disease is a hereditary disease that predominantly affects black people. It is very widespread in sub-Saharan Africa, particularly at the Lehmann “sickle belt” level, where the prevalence of the hemoglobin S involves at least 10% of the population in West Africa and can reach 40% in Central Africa. In Côte d'Ivoire, the prevalence of the hemoglobin S is about 12–14% in the general population and about 11.71% in the child population in Abidjan. On the other hand, its coexistence with other hemoglobin phenotypes such as AC (6.2%) and *β*-thalassemia (2.7%) traits may also cause composite heterogeneous sickle cell disease, e.g., SC or S/*β-*thalassemia in this study. Since 2009, sickle cell disease has been recognized as a public health problem; however, much still remains to be performed despite the progress achieved. The objective of this study is thus to promote a rapid screening for the struggling against sickle cell disease in Côte d'Ivoire. This study was carried out over 6 months (April–September 2019) and has included 336 children, of which 236 all-comers, recruited in the municipality of Treichville in Abidjan and 100 other children with already known hemoglobin phenotype followed up in the Hematology Department of the University Hospital of Treichville. Two tests were used: the HemoTypeSC™ for rapid screening and the hemoglobin electrophoresis which is the reference method used for confirming the diagnosis in the laboratory. The findings confirmed the reliability of the HemoTypeSC™ with a sensitivity and specificity at 100% for the detection of hemoglobin A, S, and C. On the other hand, this sensitivity and specificity drop to 98.2% and 99.7%, respectively, when we analyze all the 336 children together, including the cases with HbF detected by hemoglobin electrophoresis. Hence, the importance of performing certainty tests following the HemoTypeSC™ screening test in order to determine the accurate phenotypes and proportions of the types of hemoglobin. The prevalence of hemoglobin S in subgroup 1 of 236 children of all-comers was 15%. The HemoTypeSC™ is therefore reliable, inexpensive, and disposable for rapid screening and early detection of sickle cell disease in Côte d'Ivoire. The HemoTypeSC™ provides rapid detection of hemoglobin phenotypes HbAA, HbSS, HbSC, HbCC, HbAS, and HbAC.

## 1. Introduction

Sickle cell disease is a genetic condition that affects 3% of the world's population. Each year, more than 312,000 children are born with homozygous hemoglobin (SS). About 75% of them live in sub-Saharan Africa, and more than 50% of children with this condition die before the age of 5 years [1, 2]. Sickle disease was recognized as a public health problem by the WHO in 2009. In Côte d'Ivoire, studies have shown a prevalence of hemoglobin S between 12% and 14% in the general population according to Cabannes et al. [3] in 1967 and about 12% in the infant population in Abidjan with a coexistence of AC (6.2%) and AFA2 or *β*-thalassemia (2.7%) traits according to Danho Bassimbié et al. in 1988 [4]. These hemoglobin defects are most often at the origin of major sickle cell syndromes commonly found in the country.

Sickle cell disease has been recognized as a public health problem since 2009 (WHO). However, despite notable progress in the treatment of major sickle cell syndromes, screening remains insufficient in Côte d'Ivoire. Hemoglobin electrophoresis, the reference test for the diagnosis of sickle cell anemia in the laboratory, remains generally unaffordable. The Emmel test, a classic screening test, remains tedious, operator-dependent, obsolete, and not currently used. In addition, recent studies carried out in 2019 in Nigeria [5], Ghana [6], India [7], and the USA [8] have demonstrated the effectiveness of the HemoTypeSC™ test for rapid screening of sickle cell disease, particularly for the identification of abnormal genes S and C. This is a simple and inexpensive technique to handle, ready to use at the point of care.

In order to contribute to the reduction of morbidity and mortality related to sickle cell disease by promoting prevention and early management of diagnosed cases, we found it necessary to evaluate the feasibility of the HemoTypeSC™ in infant population in Abidjan.

The objective is to promote rapid screening of sickle cell disease in Côte d'Ivoire by evaluating the performance of the HemoTypeSC™ test.

## 2. Methods

We recorded 336 children in the study, including 236 children all-comers, living in the municipality of Treichville, and 100 other children with a hemoglobin phenotype already known, followed up at the Hematology Department of the Central Laboratory of the University Hospital of Treichville.

### 2.1. Type and Duration of the Study

This was a feasibility research. The study was prospective, observational, cross-sectional, descriptive, and analytical. The study was carried out over a period of six (6) months from April to September 2019.

### 2.2. Study Population

A total of 336 children were included in the study. The study population was divided into two subgroups:

  Subgroup 1 included 236 apparently healthy children all-comers, recruited in 2 schools and 1 maternal and child protection health center in the municipality of Treichville
  Subgroup 2 included 100 outpatients followed up in the hematology wards of the University Hospital of Treichville, in whom the hemoglobin phenotype was already known at the hemoglobin electrophoresis

### 2.3. Participating Sites

The venue of the study was the municipality of Treichville in the following sites:

For subgroup 1, three (3) sites were identified
  - Private primary school: Ecole Primaire Privée (EPP) “Les PETITS”
  - Secondary school: “Collège Moderne de l'Autoroute” (CMA)
  - Maternal and child protection health center: Protection Maternelle et Infantile (PMI)
∗NB: due to time constraints, newborns were not included in the final study
For subgroup 2, the participating site was the Hematology Department of the Central Laboratory of the University Hospital of Treichville

### 2.4. Selection Criteria

The inclusion criteria were as follows:

  For subgroup 1
  Any child aged 0–15, apparently healthy, recruited in the maternal and child protection center and schools with the informed consent of the parents and the consent of the children
  For subgroup 2
  Any children aged 0–15 followed up in the Hematology Department of the University Hospital of Treichville in the same period, known as sickle cell disease patient, whose hemoglobin phenotype was already identified (by hemoglobin electrophoresis).

The parents were informed and both parent and child consent was required (if applicable).

### 2.5. Tests Used

Two types of tests were proceeded: HemoTypeSC™ and hemoglobin electrophoresis. Both tests were applied in the two study subgroups.

#### 2.5.1. HemoTypeSC™

HemoTypeSC™ is a rapid test kit used in our study to determine the presence of hemoglobin A, S, and C in whole blood. It was used in first line for rapid screening of sickle cell disease in subgroup 1 and in second intention in subgroup 2 to assess its reliability.

The materials of HemoTypeSC™ Test Kit includes foil pouch containing a vial of 50 single-use test strips, a vial of 50 single-use blood sampling devices, three reusable dropper pipettes, and instructions for use.

The investigators also provide accessory materials, e.g., drinking water (not saline solutions), timer, lancing device and lancets, test vials (1.5–5 mL tubes or vials), and a rack for holding test vials.

**Figure 1:**
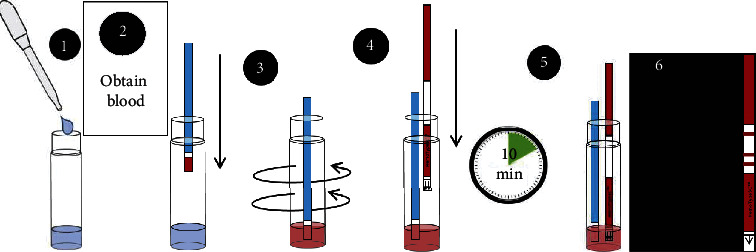
HemoTypeSC™ test running procedure.

(1) *Principle*. HemoTypeSC™ is a competitive lateral flow immunoassay incorporating monoclonal antibodies for determination of the presence of hemoglobin A, S, and C. It performs rapid detection of hemoglobin phenotypes HbAA, HbSS, HbSC, HbCC, HbAS, and HbAC.

(2) *Procedure* (Figure 1). This procedure encompasses 6 steps (cf. instructions for use form).

(1) Using dropper pipette, add six (6) drops of water to the test vial. Place the test vial in a compatible rack.
(2) Open the vial of blood sampling devices, remove one blood sampling device, and reclose the vial. Collect blood sample (a small drop is sufficient, 1-2 microliters). Touch the white pad of the blood sampling device to collect blood sample, until the white pad absorbs the blood droplet. Ensure that the entire white pad has turned red.
(3) Insert the blood sampling device into test vial with water and swirl to mix
  - Sufficient swirling is essential for blood to be properly transferred into the test vial
  - Check visually to ensure that water has become pink or light red in color
  - Leave the blood sampling device in the test vial after swirling
(4) Open the vial of test strips, remove one test strip, and reclose the vial. Insert HemoTypeSC™ test strip into the test vial with arrows pointing down
(5) Wait 10 minutes
(6) Take HemoTypeSC™ test strip out of the test vial and read results. Compare test strip to results chart on reverse side of the document for reference

(3) *Reading and Interpretation of Results.* Red lines may appear at each of three hemoglobin variant specific locations (HbA, HbS, and HbC) and a control location and are compared with the chart for interpretation:

- The presence of a line on the strips indicates the absence of the hemoglobin variant in the blood sample
- The absence of a line on the strips indicates the presence of the hemoglobin variant in the blood sample

If the control line is absent, result is invalid; the test is invalid and must be repeated (note: it is important to have a good source of light for identifying the hemoglobin migration lines on the strip).

*(4) Training of Operators*. Information sessions and training of test protocols were provided to operators by technicians from Distrilabo Africa, an IVD products distribution agency, at the start of the study. The training tools were based on the instructions for use and the demonstration video. The HemoTypeSC™ test is an easy technique to perform which does not require intensive training. The operators were lab personnel and research assistants. However, trained clinical staff can also perform the HemoTypeSC™ test.

#### 2.5.2. Hemoglobin Electrophoresis (Helena Method)

This is the classic agarose gel test, used routinely in the Central Laboratory of the University Hospital of Treichville for the biological diagnosis of sickle cell anemia. It was used in this study as a second-line reference method in children in subgroup 1 to confirm the phenotype of hemoglobin and determine the percentage of different types of hemoglobin. For subgroup 2 of sick children, it was also used for checking their specific hemoglobin phenotypes.

### 2.6. Ethical Considerations

The study protocol was validated by the Ethics Committee of the Ministry of Health. Respect for the principle of confidentiality has been applied. Parents' informed consent to their children's participation in the study was obtained before their inclusion in the study. The referral of cases of major sickle cell syndromes to the hematology department has been recommended, as well as genetic counseling for hemoglobin defect traits. Personal hemoglobin type cards recording the hemoglobin phenotype and blood group of all children have also been established.

### 2.7. Data Management

The data were collected in paper form, entered under EPIDATA 3.1, and saved on an external hard disk. Data provided were analyzed under STATA 14.1.

## 3. Main Findings

### 3.1. Study Sample

A total of 336 were children selected according to a random sampling process at 4 participating sites level (100 from the Hematology Department of the University Hospital of Treichville and 236 from the Maternal and Child Protection Health Center (PMI), the private primary school “EPP LES PETITS,” and secondary school (College Moderne de l' Autoroute (CMA)) in the municipality of Treichville (cf.  Table 1)).

The subgroup refers to all-comers children and represents 70% of the total study population.

The subgroup 2 refers to the sick children recruited from the Hematology Department of the University Hospital of Treichville (30%) in order to assess the reliability of the HemoTypeSC™ screening test.

### 3.2. Sociodemographic Features

The HemoTypeSC™ test was assessed on the 336 children from the 4 participating sites (Table 1). The sex distribution (Table 2) shows female predominance in both groups (female (54%); male (46%)) that gives an overall sex ratio of 0.84. The sex ratio for is, respectively, 0.81 for subgroup 1 and 0.89 for subgroup 2. The age distribution shows a predominance of the age group from 6 to 10 years (42%) in both subgroups (Table 3).

Both subgroups have the same age structure. The age group from 6 to 10 years is predominant.

### 3.3. Analysis of the Sensitivity and Specificity of the HemoTypeSC™ for the Detection of Hemoglobin A, S, and C

HemoTypeSC™ test combines independent immunoassays with monoclonal antibodies specific for each of the 3 types of hemoglobin A, S, and C. Therefore, the sensitivity and specificity of HemoTypeSC™ were first calculated for the detection of each type of hemoglobin (Table 4):

- For HbA, the HemoTypeSC™ test had 100% sensitivity and specificity: this means 239 positive results for HbA on all 239 samples containing HbA and 97 negative results on all 97 samples not containing HbA
- For HbS and HbC, the HemoTypeSC™ test also had, respectively, 100% sensitivity and specificity

The values summarized in Table 4 indicate the results of the HemoTypeSC™ test compared to the reference method (electrophoresis of hemoglobin), taking into account both subgroups.

- Any specimen that was heterozygous or homozygous for each particular variant of Hb was considered to be true-positive for that variant
- Any specimen without the particular hemoglobin was considered to be true-negative for that hemoglobin, regardless of the other hemoglobin variants present

### 3.4. Analysis of the Hemoglobin Phenotypes

The findings showed the following phenotypes:

(i) Homozygous phenotypes
  1. HbAA (wild type or normal)
  2. HbSS (homozygous sickle cell disease)
  3. HbCC (hemoglobin C disease)
(ii) Heterozygous phenotypes
  1. HbAS (sickle cell trait)
  2. HbAC (AC trait)
  3. HbSC (composite heterozygous sickle cell disease SC)

In this study, the HemoTypeSC™ test can correctly detect the phenotypes HbSS, HbCC, HbSC, and HbAC with no false-positive results, showing thus a sensitivity and specificity of 100%, respectively.

(i) HemoTypeSC™ fails to detect HbF and other hemoglobin variants other than HbS, HbC, and HbA, whereas hemoglobin electrophoresis helped to detect
  1. 2 cases of HbSFA2 (composite S/*β*0-thalassemia-sickle cell disease)
  2. 4 cases of HbSAFA2 (composite S/*β*+ thalassemia-sickle cell disease)

Consequently, the sensitivity and the positive predictive value (PPV) were 0%, respectively, for SAFA2 (S/*β*+ thalassemia) and SFA2 (S/*β*0-thalassemia).

The results of the electrophoresis of hemoglobin were compared to those of the HemoTypeSC™ test:

- SFA2 phenotype (S/*β*0-thalassemia) produces the same result as SS at HemoTypeSC™
- SAFA2 phenotype (S/*β*+ thalassemia) produces the same result as AS at HemoTypeSC™

Thus, the sensitivity and specificity of HemoTypeSC™ drop, respectively, to 98.2% and 99.7% as the HemoTypeSC™ test does not detect HbF (Table 5).

### 3.5. Analysis of the Prevalence of Sickle Cell Disease in Subgroup 1 (All-Comers)

A total of 35 children out of 236 children in subgroup 1 recruited from schools and health centers had abnormal hemoglobin, that is, an overall prevalence of 14.8%. The S gene is present in 12% and the C gene in 2.8% of the cases. The electrophoresis of hemoglobin made it possible to bring out different phenotypes (Table 6).

## 4. Discussion

Sickle cell anemia is one of the most common genetic diseases in Côte d'Ivoire. In this study, the prevalence of the S gene is 12% in the population of 236 all-comers children. This prevalence rate is close to those reported by Cabannes et al. in 1967 [3] and Danho Bassimbié et al. [4] in 1988, estimated as 11.7% in child population in Abidjan as well. This prevalence rate seems stable. Promoting early screening in Côte d'Ivoire seems to be one of the best strategies to help prevent morbidity and mortality related to sickle cell disease.

According to previous data reported in the literature, early diagnosis and management of sickle cell disease would prevent 70% of deaths related to sickle cell disease [8]. Early screening of children allows children with sickle cell disease to be identified before they have symptoms or complications. These children can then be followed up regularly with comprehensive care and timely management to reduce morbidity and mortality [9–11].

Moreover, some main obstacles to implementing sickle cell disease screening programs are the high cost of conventional diagnostic methods, the lack of adequate equipment, and insufficient funding. Basically, it becomes imperative to promote rapid, inexpensive, and accurate screening methods such as HemoTypeSC™ in Côte d'Ivoire. This method has already been assessed in other countries such as Nigeria, Ghana, and India [9–11].

In our study, we thus assessed the feasibility of the HemoTypeSC™ test for screening sickle cell disease as a “Point Of Care” device in the municipality of Treichville in Côte d'Ivoire.

Our findings showed that HemoTypeSC™ is a reliable, efficient, and accurate test that detects hemoglobin A, S, and C with 100% sensitivity and specificity (Table 4). On the other hand, the sensitivity and specificity of the HemoTypeSC™ drop to 98.2% and 99.7%, respectively, when the cases of S/*β*0-thalassemia and S/*β*+ thalassemia detected with hemoglobin electrophoresis are included in the analysis. As stated in the principle of HemoTypeSC™, the sensitivity for HbF is 0%. Thus, it did not detect hemoglobin F.

Previous studies also confirmed our findings [5–7]. Recent studies using the HemoTypeSC™ in low-resource study centers in sub-Saharan Africa also showed a degree of accuracy of 100% in the detection of genes A, S, and C for newborns blood samples that contain more than 80% of HbF [6, 12].

**Figure 2:**
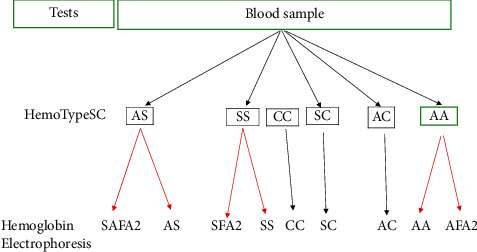
Decision-making algorithm for the diagnosis of sickle cell disease.

Therefore, HemoTypeSC™ can be used in Côte d'Ivoire as a screening test for sickle cell disease. This is a rapid and reliable test to detect the AA, AS, AC, SS, SC, and CC phenotypes [5–8]. HemoTypeSC™, being a qualitative and not quantitative screening test, determines the presence or absence of HbA, HbS, and HbC. It can be completed by a certainty test which helps bring out other hemoglobin such as fetal Hb (HbF) not detected by the rapid screening test. In fact, phenotype HbSFA2 or S/*β*0-thalassemia will produce result as HbSS at HemoTypeSC™ and phenotype SAFA2 or S/*β*+ thalassemia will produce AS. So, the decision-making algorithm is recommended for detection of hemoglobin S and C as shown in Figure 2. This algorithm encompasses 3 levels of decision.  ^*∗*^NB, other hemoglobin variants, e.g., HbF, HbD, HbE, will produce the same HemoTypeSC™ result as HbA.

### 4.1. Level 1

HemoTypeSC™ confirms the presence of hemoglobin S, C, and A, but it does not detects HbF and other variants.

Hence, there is a need to carry out a secondary certainty test (e.g., hemoglobin electrophoresis) to confirm the definitive phenotype as follows:

- HbAS may produce HbAS or HbSAFA2 (S/*β*+ thalassemia)
- HbSS may produce HbSS or HbSFA2 (S/*β*0-thalassemia).
- HbAA may produce HbAA or HbAFA2

### 4.2. Level 2

When the HemoTypeSC test produces HbAC, HbCC, and H phenotypes, this is 100% reliable.

### 4.3. Level 3

When the HemoTypeSC test produces HbAA phenotype, it also recommended to proceed a certainty test that specifies the hemoglobin phenotype HbAA as it may produce HbAA or HbAF (other rare hemoglobin variants).

Basically, HemoTypeSC™ is a reliable and low cost screening test to detect the presence and the percentage of the S gene and other abnormal hemoglobin such as C gene. A certainty test is thus recommended for further analysis and for the determination of the percentage of hemoglobin.

Based on these findings, we thus recommend the use of HemoTypeSC™ for sickle disease screening in countries of high prevalence and in low-income settings, e.g., Côte d'Ivoire. It must be completed by a certainty test for further investigation. We therefore propose the above algorithm (Figure 1) to guide the diagnosis of sickle cell anemia. This algorithm may also be oriented by clinical signs.

HemoTypeSC™ helps to determine the hemoglobin S status among the general population, infant, newborns, and pregnant women as well. It can contribute to the prevention of sickle cell disease through sensitization on genetic counseling, prenuptial, prenatal, and neonatal screening. Prevention does indeed reduce the high prevalence of sickle cell disease. Early diagnosis and comprehensive management of major sickle cell disease syndromes do really improve the prognosis of children and persons living with major sickle cell syndromes.

Besides the HemoTypeSC™, there are also other points of care screening devices such as Sickledex and Sicklecheck [13–18]. Despite these notable advances in diagnosis for sickle cell disease, these tests require specific instrumentation related to their own procedures. Some constraints to their use in countries with limited financial resources are due to the fact that these tests failed to detect sickle cell disease in the presence of severe anemia. This results in false-positives. The reagents also had a relatively short shelf life which required regular supply. Besides the technical performance, the HemoTypeSC™ has also socioeconomic advantages as it is a low cost test, e.g., USS5 compared to USS30 for hemoglobin electrophoresis.

## 5. Conclusion

HemoTypeSC™ is a rapid and low cost disposal for the detection of hemoglobin A, S, and C. One just needs a small drop of whole blood. No refrigeration, instrumentation, or energy sources are required. HemoTypeSC™ does not require extensive operator training. It provides an accurate and reliable diagnosis for abnormal hemoglobin S and C in children, newborns, pregnant women, and the general population. Therefore, we recommend the use of HemoTypeSC™ in countries of high prevalence of sickle cell disease, e.g., Côte d'Ivoire as first the line screening test for sickle cell disease. In addition, we recommend the hemoglobin electrophoresis as a certainty test for further investigation. We thus proposed a decision-making algorithm for diagnosis of sickle cell disease. The HemoTypeSC™ screening must be popularized according to the WHO recommendations [17], as regards to the African strategy to better fight against sickle cell disease. This involves primary prevention not only through early prenuptial, prenatal, neonatal screening but also mass assessment, with the commitment of all key stakeholders.

## Figures and Tables

**Table 1:** Distribution of the study population according to the sampling site.

Sites	*N*	%
Subgroup 1 (*n* = 236)		
EPP LES PETITS^*∗*^	124	52.5
CMA^*∗∗*^	67	28.4
PMI^*∗∗∗*^	45	19.1
S/total	236	100.0

Subgroup 2 (*n* = 100)		
Hematology department, CHU T	100	100
*S*/total	100	100.0
Total	336	100

Chi-square = 0; 12 ddl = 1; *p*=0.72; there is no statistically significant difference in 2 subgroups.  ^*∗*^EPP LES PETITS, Ecole Primaire Privée LES PETITS (private primary school). ^*∗∗*^CMA, ‘'Collège Moderne de l'Autoroute (modern secondary school). ^*∗∗∗*^PMI, Protection Maternelle et Infantile (maternal and child protection health center). Hematology department, CHU T, Service d'hématologie, Center Hospitalo-universitaires de Treichville (University Hospital of Treichville).

**Table 2:** Distribution by sex.

Sex	Sub-group 1		Sub-group 2		Total	
	*N*	%	*N*	%	*N*	%
M	106	45	47	47	153	46

F	130	55	53	53	183	54

Total	236	100	100	100	336	100

Sex ratio	0.81		0.89		0.84	

**Table 3:** Distribution of the study population by age.

Age	*N*	%
<5 years	99	30
6–10 years	141	42
11–15 years	96	28
Total	336	100

**Table 4:** Analysis of the sensitivity and specificity of the HemoTypeSC™ test for the detection of hemoglobin A, S, and C.

Type of Hb	Sensitivity (TP/(TP + FN))		Specificity (TN/(TN + FN))		PPV (TP/(TP + FP))		NPV (TN/(TN + FN))	
A	239/239	100%	97/97	100%	239/239	100%	97/97	100%
S	126/126	100%	110/110	100%	126/126	100%	110/110	100%
C	28/28	100%	308/308	100%	28/28	100%	308/308	100%
Total	393/393	100%	515/515	100%	393/393	100%	515/515	100%

FP, false-positives; FN, false-negatives; TP, true-positives; TN, true-negatives; PPV, positive-predictive value; NPV, negative-predictive value.

**Table 5:** Analysis of the hemoglobin phenotypes by HemoTypeSC™.

Phenotype Hb	Sensitivity (TP/(TP + FN))		Specificity (TN/(TN + FN))		PPV (TP/(TP + FP))		NPV (TN/(TN + FN))	
AA	201/201	100%	135/135	100%	201/201	100%	135/135	100%
AS	29/29	100%	307/307	100%	29/31	93.5%	307/307	100%
SS	72/72	100%	263/263	100%	72/76	94.7%	263/263	100%
SC	20/20	100%	316/316	100%	20/20	100%	316/316	100%
AC	7/7	100%	329/329	100%	7/7	100%	329/329	100%
CC	1/1	100%	335/335	100%	1/1	100%	335/335	100%
SAFA2	0/2	0%	334/336	99.4%	0/2	0%	334/336	99.4%
SFA2	0/4	0%	332/336	98.8%	0/4	0%	332/336	98.8%
Total	330/336	98.2%	235/235	99.7%	330/342	96.5%	2351/2357	99.7%

FP, false-positive; FN, false-negative; TP, true-positive; TN, true-negative; PPV, positive predictive value; NPV, negative predictive value.

**Table 6:** Distribution of the population of subgroup 1 according to the hemoglobin phenotype.

Hemoglobin phenotype	Effect	
	*N*	%
AA	201	85.2
AS ^*∗*^	26	11.2
AC	6	2.4
SSFA2^*∗∗*^	1	0.4
SFA2^*∗*^	1	0.4
CC	1	0.4
Total	236	100

NB: The prevalence of the S gene is 12% in the all-comers children-subgroup 1 (236 children).

## Data Availability

The laboratory and epidemiological data used to support the findings of this study are available from the corresponding author upon request.

## References

[1] Piel F. B., Patil A. P., Howes R. E. (2013). Global epidemiology of sickle haemoglobin in neonates: a contemporary geostatistical model-based map and population estimates. *The Lancet*.

[2] Grosse S. D., Odame I., Atrash H. K., Amendah D. D., Piel F. B., Williams T. N. (2011). Sickle cell disease in Africa. *American Journal of Preventive Medicine*.

[3] Cabannes R., Baba S. Y., Schmitt-Beurrie A. (1967). Etude des hémoglobinopathies en côte d’ivoire. *Medicale d’Afrique Noire*.

[4] Danho Bassimbie J., Fabritius H., Sangare A., Abissey S. A., Tea D., Cabannes R. (1988). Prévalence des hémoglobines anormales dans une population infantile à Abidjan. *Pan African Medical Journal*.

[5] Nnodu O., Isa H., Nwegbu M. (2019). HemoTypeSC, a low-cost point-of-care testing device for sickle cell disease: promises and challenges. *Blood Cells, Molecules and Disease*.

[6] Steele C., Sinski A., Asibey J. (2019). Point-of-care screening for sickle cell disease in low-resource settings: a multi-center evaluation of HemoTypeSC, a novel rapid test. *American Journal of Hematology*.

[7] Mukherjee M. B., Colah R. B., Mehta P. R. (2020). Multicenter evaluation of HemoTypeSC as a point-of-care sickle cell disease rapid diagnostic test for newborns and adults across India. *American Journal of Clinical Pathology*.

[8] Quinn C. T., Paniagua M. C., DiNello R. K., Panchal A., Geisberg M. (2016). A rapid, inexpensive and disposable point-of-care blood test for sickle cell disease using novel, highly specific monoclonal antibodies. *British Journal of Haematology*.

[9] Telfer P., Coen P., Chakravorty S. (2007). Clinical outcomes in children with sickle cell disease living in England: a neonatal cohort in east London. *Haematologica*.

[10] Tshilolo L., Kafando E., Sawadogo M. (2008). Neonatal screening and clinical care programmes for sickle cell disorders in sub-Saharan Africa: lessons from pilot studies. *Public Health*.

[11] Quinn C. T., Rogers Z. R., McCavit T. L., Buchanan G. R. (2010). Improved survival of children and adolescents with sickle cell disease. *Blood*.

[12] Nankanja R., Kadhumbula S., Tagoola A., Geisberg M, Serrao E, Balyegyusa S (2019). HemoTypeSC demonstrates >99% field accuracy in a sickle cell disease screening initiative in children of southeastern Uganda. *American Journal of Hematology*.

[13] Hara S. (1973). Reliability and modification of Sickledex test. *Journal of the National Medical Association*.

[14] Kumar A. A., Patton M. R., Hennek J. W. (2014). Density-based separation in multiphase systems provides a simple method to identify sickle cell disease. *Proceedings of the National Academy of Sciences*.

[15] Yang X., Kanter J., Piety N. Z., Benton M. S., Vignes S. M., Shevkoplyas S. S. (2013). A simple, rapid, low-cost diagnostic test for sickle cell disease. *Lab on a Chip*.

[16] Kanter J., Telen M. J., Hoppe C. (2015). Validation of a novel point of care testing device for sickle cell disease. *BMC Medicine*.

[17] World Health Organization (2010). *Sickle Cell Disease: A Strategy for the WHO Africa Region*.

[18] Smart L. R., Ambrose E. E., Raphael K. C. (2017). Simultaneous point-of-care detection of anemia and sickle cell disease in Tanzania: the RAPID study. *Annals of Hematology*.

